# Sporadic Form of Recurrent Atrial Myxoma: The Blob Strikes Back

**DOI:** 10.7759/cureus.9745

**Published:** 2020-08-14

**Authors:** Rupinder Buttar, Ryan Hoefen, Matthew Funderburk, Enzo Fallone, Bipul Baibhav

**Affiliations:** 1 Cardiology, Rochester Regional health, Rochester, USA; 2 Cardiology, Sands-Constellation Heart Institute, Rochester Regional Health, Rochester, USA; 3 Cardiology, Rochester Regional Health, Rochester, USA; 4 Pathology, Rochester Regional Health, Rochester, USA

**Keywords:** recurrent myxoma, atrial myxoma, transthoracic echocardiogram, mri cardiac

## Abstract

Cardiac myxoma is a benign neoplasm composed of stellate to plump, cytologically bland mesenchymal cells set in a myxoid stroma. Although benign, as they can lead to severe complications, they are often removed surgically. A 39-year-old female presented with a chief complaint of generalized fatigue. Patient had a history of a large 7cm x 2.5cm left atrial myxoma resected at the age of 32 years after she presented with symptoms of dyspnea on exertion. The dyspnea was due to prolapse of the mass through the mitral valve during diastole, leading to functional severe mitral stenosis. The mass was resected with clear margins confirmed on biopsy. On physical examination, heart rate was regular with no murmurs. No signs of congestive heart failure were noted. A 2D echo revealed a mobile structure in the left atrium along with mild mitral regurgitation. Cardiac MRI showed a 21mm x 9mm well defined, pedunculated, mobile mass in the left atrium arising from inter-atrial septum. The mass was hyperintense on T2 weighted images with patchy delayed hyper-enhancement consistent with recurrence of a myxoma. The patient underwent a repeat median sternotomy with the removal of left atrial mass and repair of atrial septum with hemashield patch. The mass was sent for pathological evaluation confirming the diagnosis of recurrent myxoma. On genetic testing, patient tested negative for mutations in PRKAR1A gene (mutated in up to 60%-80% cases with Carney complex), MEN1, RET and sarcoma (TP53) genes. Cardiac myxomas are rare primary benign tumors of the heart with a small recurrence rate. Follow-up studies have rarely reported recurrences after complete resection. However, in our case not only did the patient have the sporadic form of myxoma with recurrence, but it also occurred within three years of the previous resection despite complete removal with clear margins.

## Introduction

Cardiac myxomas are the most common primary benign tumors of the heart. The World Health Organization (WHO) defines a cardiac myxoma as a neoplasm composed of stellate to plump, cytologically bland mesenchymal cells set in a myxoid stroma. They usually arise in the atrial chambers with a greater predisposition for left atrial chamber than the right. Although these tumors are benign, they can lead to complications such as mitral valve obstruction or systemic embolism. As a result, they are often removed surgically.

Sporadic cases have a very low recurrence rate of 1%-3 %, whereas patients with familial forms and complex forms have higher recurrence ranging from 12%-22% [1].

## Case presentation

**Figure 1:**
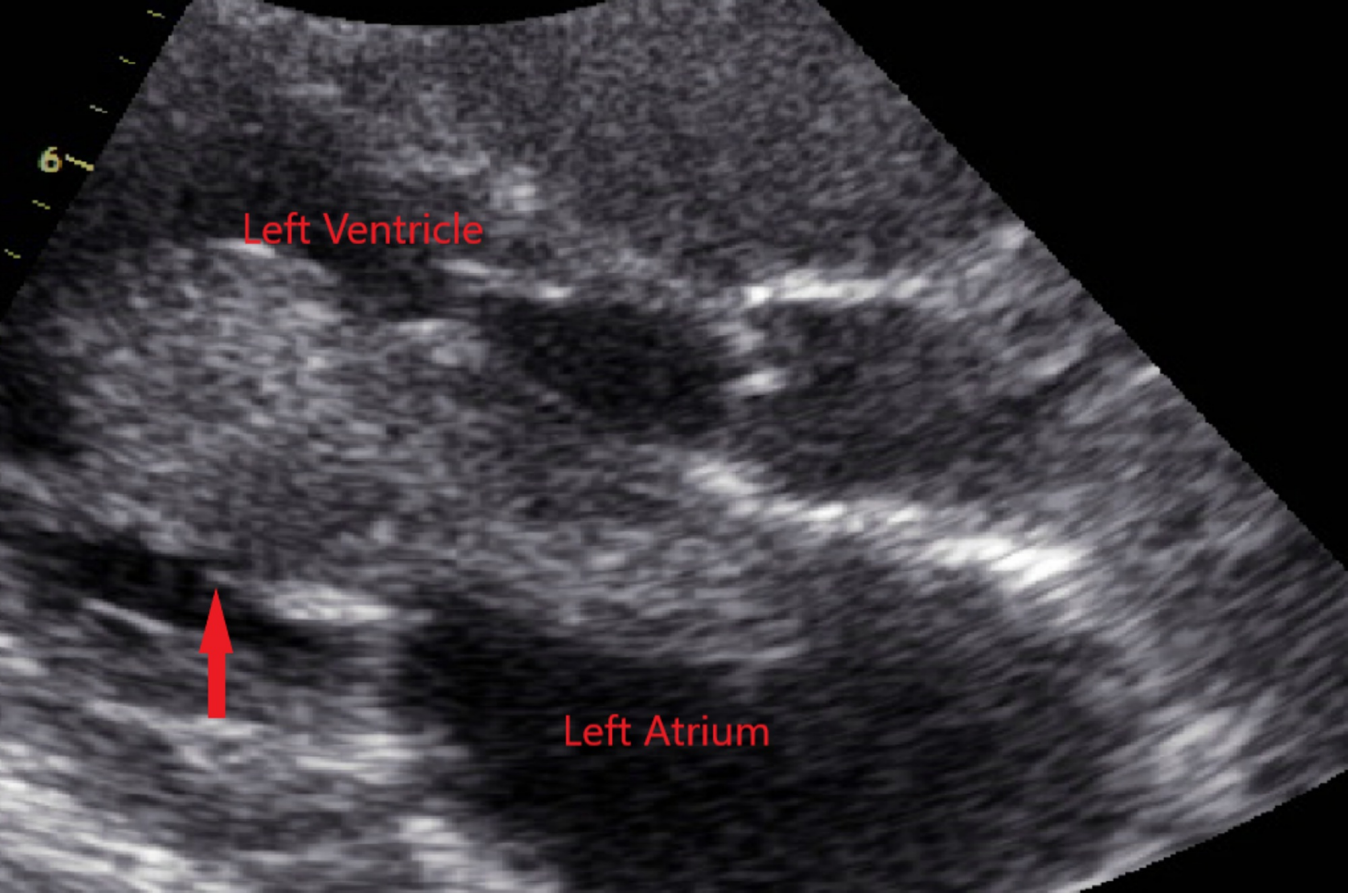
Parasternal long axis on transthoracic echocardiogram showing prolapse of the large left atrial mass into the left ventricle

A 39-year-old woman presented with a chief complaint of generalized fatigue. She denied any fevers, weight loss, chest pain, and shortness of breath, orthopnea, edema, syncope or light-headedness. She had a history of left atrial myoma that was resected three years ago. The myxoma was diagnosed after she presented with acute hypoxic respiratory failure secondary to acute pulmonary edema. She was found to have a 7.0 cm x 2.5 cm left atrial myxoma connected to the interatrial septum on echocardiogram as seen in Figure 1.

**Figure 2:**
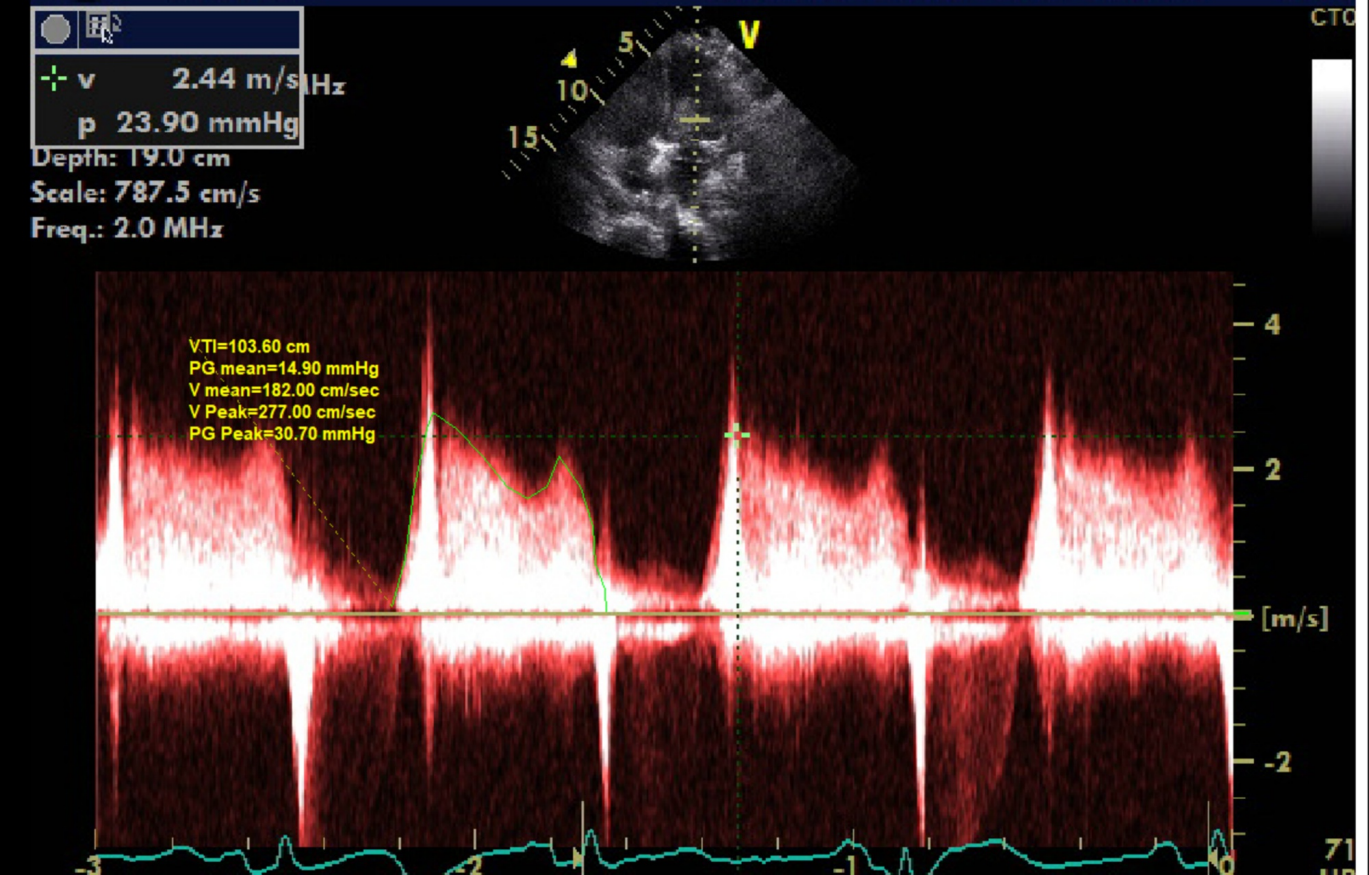
Severe functional mitral stenosis related to prolapse of left atrial myxoma into the left ventricle during diastole

There was severe functional mitral valve stenosis due to the prolapse of the left atrial mass through the mitral valve during diastole as seen in Figure 2.

The biopsy was consistent with a myxoma with clear margins. Patient’s past medical history was also significant for Ewing’s sarcoma, pelvic sarcoma and parathyroid adenomas. There was no family history for myxomas or other endocrinological tumors.

**Figure 3:**
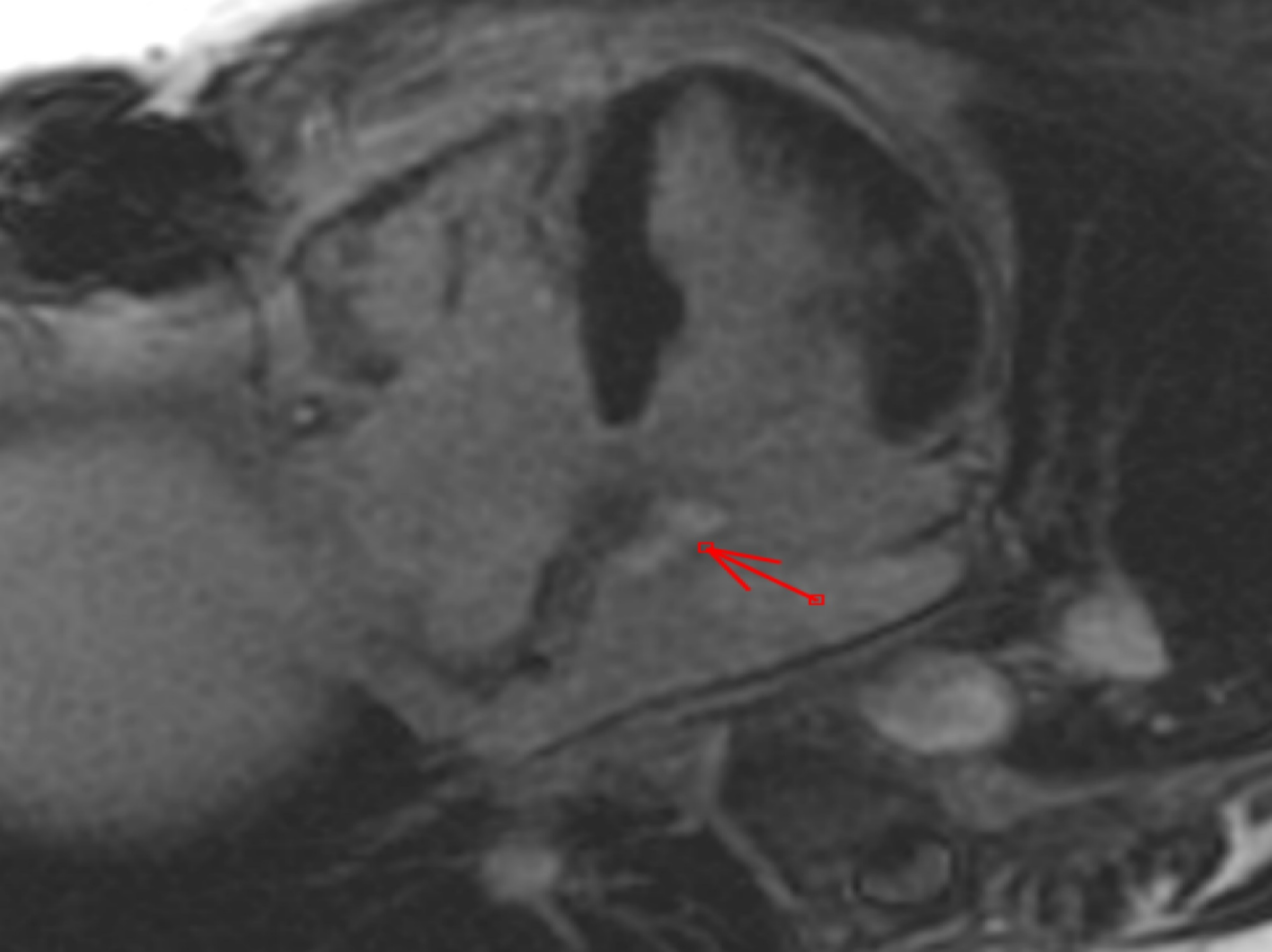
Post-contrast four-chamber image showing late gadolinium enhancement of the left atrial mass consistent with fibrosis

**Figure 4:**
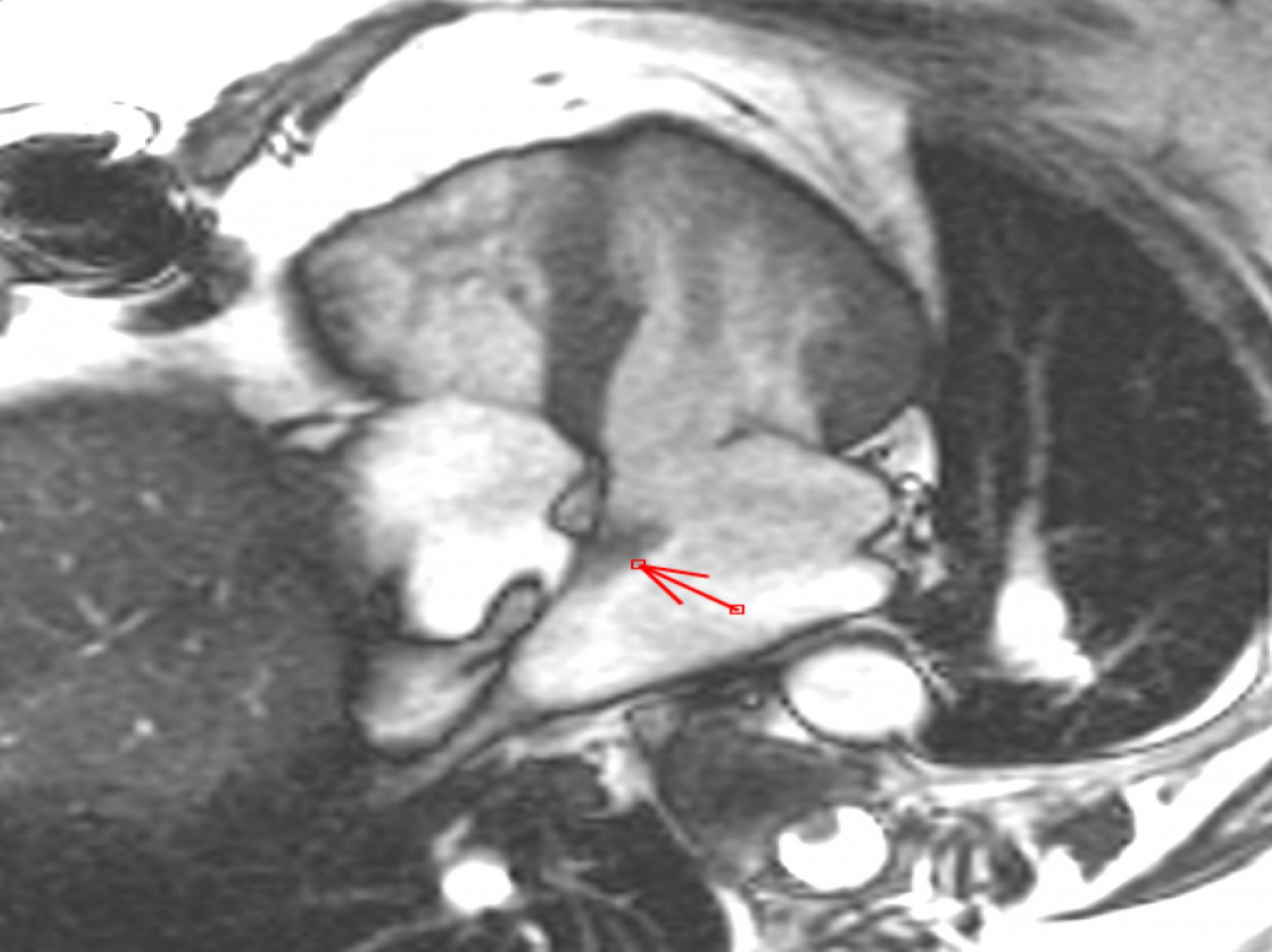
Chamber SSFP T2 image showing left atrial mass attached to the inter-atrial septum SSFP, steady-state free precession

On examination, patient had normal heart rate with regular rhythm. No murmurs were appreciated. No signs of congestive heart failure were noted. An echocardiogram revealed a mobile structure in the left atrium attached to the inter-atrial septum. Further evaluation by cardiac MRI showed a 2.1 cm x 0.9 cm well defined, pedunculated, mobile mass in the left atrium arising from inter-atrial septum. The mass was hyperintense on T2-weighted imaging. The mass demonstrated patchy delayed gadolinium enhancement on postcontrast images. Based on location and MRI characteristics the most likely diagnosis was the recurrence of myxoma (Figures 3, 4)

**Figure 5:**
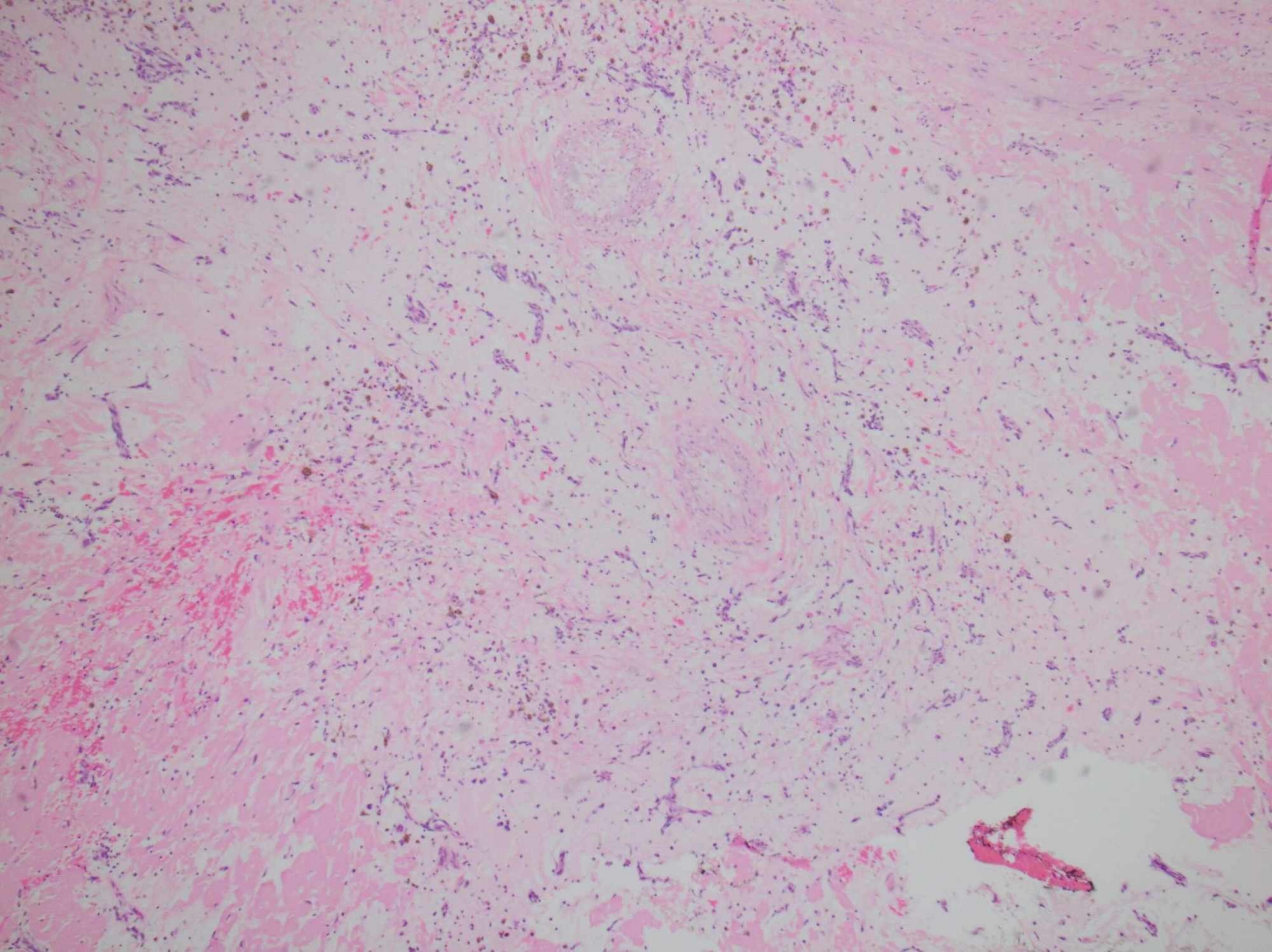
Hypocellular myxoid mass with many small capillaries (40x)

**Figure 6:**
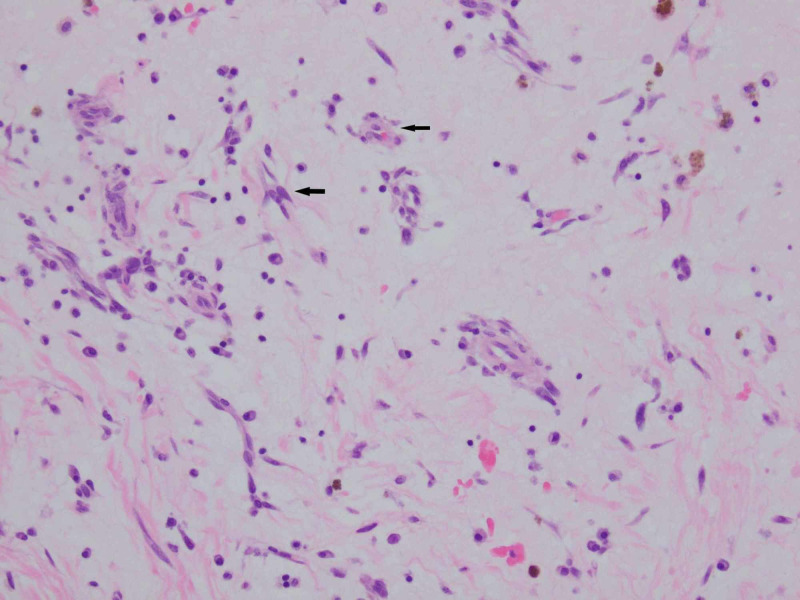
Spindled and stellate-shaped cells (solid arrows) deposited in an extensive myxoid stroma with chronic inflammatory cells (200x)

The patient had a repeat median sternotomy with removal of left atrial mass and repair of atrial septum with hemashield patch. The mass was sent for pathological evaluation, which showed soft tissue with myxoid changes along with chronic inflammation and hemosiderin, confirming the diagnosis of recurrent myoma (Figures 5, 6). No operative or post-operative complications were noted.

On follow-up, the patient was asymptomatic and in sinus rhythm. Given her history of recurrent myxomas and multiple endocrinological tumors, she was referred for genetic testing, which was negative for PRKAR1A gene, which is mutated in up to 60%-80% cases with Carney complex. She also tested negative for germline susceptibility to endocrine disorders including MEN1, RET and sarcoma (TP53) genes. As no clear cut guidelines are available regarding follow-up in such cases, the plan was to obtain at least an annual transthoracic echocardiogram to evaluate for recurrence of the tumor.

## Discussion

Cardiac myxomas are benign tumors of the heart that most commonly arise from the left atrial chamber. Although the recurrence rates are extremely low in the general population, the rate is significantly higher in patients with familial forms and complex syndromes.

One such complex syndrome is Carney complex, a rare familial syndrome associated with recurrent atrial myxomas along with endocrine and non -endocrine tumors, abnormal skin pigmentation and schwannomas [2]. Although PRKAR1A gene has been found mutated in up to 80% of the patients with Carney syndrome, no association has been found between the gene and sporadic myxomas. On the other hand, most of these cases involved chromosomal regions 12p1 and 17p1. Several mechanisms have been suggested for these recurrences [3,4]. These include : 

1. Incomplete resection leading to a regrowth.

2. Familial predisposition for recurrence.

3. Implantation of embolic fragments of the original tumor in the myocardium spontaneously or due to a previous surgery.

4. Recurrence occurring from a pretumor focus present in another part of the myocardium. 

5. Malignant transformation of myxoma.

Atrial myxoma can present with a wide variety of presentations depending on location, mobility and tumor size. The symptoms also vary depending from being asymptomatic to constitutional symptoms including anemia, fever, cough, weight loss and headache, to embolization and hemodynamic consequences. The constitutional symptoms are linked to inflammatory markers produced by the body as well as the tumor itself including IL-6 [5,6]. The myxomas may also embolize leading to a range of manifestation including pulmonary embolism, mesenteric ischemia, strokes and even acute coronary syndrome. Various factors that have been linked to these embolic events in myxomas include tumor size, location and macroscopic appearance [7]. Hemodynamic symptoms are the most clinical presentation in patients with myxomas. These can range from syncope, palpitations, atrial fibrillation, heart failure to sudden cardiac death [5].

Atrial myxomas are usually diagnosed on transthoracic echocardiography (TTE), which provides information regarding structure, location and hemodynamics in case of myxomas. TTE may be followed by trans-esophageal echocardiography and cardiac MRI in cases where the TTE evaluation is limited by poor diagnostic windows and poor characterization of the mass.

The treatment of choice for myxomas remains surgical excision since the report of the first resection by Swedish cardiac surgeon Clarence Crafoord and his team in 1954 [8]. Although different techniques have been proposed for the resection, the most common approach is the median sternotomy with distal ascending aorta and bicaval cannulation.

Even though myxomas seldom recur after complete removal as described earlier, most recurrences are seen in the first 10 years.

Our patient presented with atrial myxoma for the first time at the age of 36 years. The mean age reported is between 42-66 years. Younger cases are usually seen in familial forms. However, in our case not only did the patient have sporadic form of myxoma with recurrence, but it also occurred within three years of previous event despite complete surgical resection.

## Conclusions

Atrial myxomas are the most common tumors arising in the heart. Although benign, they can lead to a wide variety of symptoms including embolization and heart failure. As a result, they are usually removed by surgical resection. Although rare, recurrences can be seen in familial cases.
